# Gender-Specific Related Factors for Suicidal Ideation During COVID-19 Pandemic Lockdown Among 5,175 Chinese Adolescents

**DOI:** 10.3389/fpubh.2022.810101

**Published:** 2022-03-02

**Authors:** Jin Zhu, Baohua Li, Fengcheng Hao, Linlin Luo, Song Yue, Jinguo Zhai, Min Chen, Yan Liu, Debiao Liu, JianLi Wang

**Affiliations:** ^1^Shandong Key Laboratory of Behavioral Medicine, School of Mental Health, Jining Medical University, Jining, China; ^2^Shandong Collaborative Innovation Center for Diagnosis and Treatment and Behavioral Interventions of Mental Disorders, Institute of Mental Health, Jining Medical University, Jining, China; ^3^Center of Evidence-Based Medicine, Jining Medical University, Jining, China; ^4^School of Mental Health, Jining Medical University, Jining, China; ^5^Daizhuang Hospital of Shandong Province, Jining, China; ^6^Zoucheng People's Hospital, Zoucheng, China; ^7^Department of Pathology, School of Basic Medicine, Weifang Medical University, Weifang, China; ^8^Department of Community Health and Epidemiology, Faculty of Medicine, Dalhousie University, Halifax, NS, Canada

**Keywords:** adolescents, COVID-19 pandemic lockdown, suicidal ideation, gender differences, predictive factor

## Abstract

**Background:**

Suicide was an urgent issue during the pandemic period in adolescents. However, few studies were focused on suicide during the coronavirus disease 2019 (COVID-19) pandemic lockdown.

**Methods:**

An online survey was conducted among 5,175 Chinese adolescents from June 9th to 29th in 2020 to investigate the prevalence of suicidal ideation (SI) during COVID-19 pandemic lockdown. A gender-specific stepwise logistic regression model was used. All analyses were performed with STATA 15.0.

**Results:**

About 3% of the participants had reported having SI during the COVID-19 pandemic lockdown period. The prevalence of female SI (3.64%, 95% CI: 2.97–4.45%) was higher than that of males (2.39%, 95% CI: 1.88–3.05%) (χ^2^ = 6.87, *p* = 0.009). Quarreling with parents [odds ratio (OR) = 9.73, 95% CI: 5.38–17.59], insomnia (OR = 5.28, 95% CI: 2.81–9.93), previous suicide attempt history (OR = 3.68, 95% CI: 1.69–8.03), previous SI history (OR = 2.81, 95% CI: 1.30–6.06), and feeling depressed during pandemic lockdown (OR = 2.26, 95% CI: 1.22–4.18) were positively associated with the males' SI. However, having emptiness inside (OR = 4.39, 95% CI: 2.19–8.79), quarreling with parents (OR = 3.72, 95% CI: 2.16–6.41), insomnia (OR = 3.28, 95% CI: 1.85–5.80), feeling anxious (OR = 2.62, 95% CI: 1.46–4.70), and longing for father's emotional warmth (OR = 0.38, 0.20–0.72) were associated mostly with females' SI.

**Conclusions:**

Female adolescents, who felt emptiness from their families and their fathers' emotional warmth, were at much higher risk of having SI during COVID-19 lockdown. We must specify a suicide prevention policy and interventions for adolescents in the pandemic crisis based on gender gaps.

## Introduction

Suicide has become the second leading cause of death among young people aged 15 to 29 (1, 2), with a rate of 4.57 per 100,000 (3). It is well recognized that suicidal ideation (SI) is an important predictor of suicide death (4). A meta-analysis showed that the prevalence of SI was 28% in adolescents up to 25 years (5). Factors such as negative life experiences (e.g., childhood abuse, bullying), unhealthy behaviors (e.g., smoking, alcohol abuse, poor parental relationships), psychological factors (e.g., low emotional support, low self-esteem, impulsivity), and physical illnesses (e.g., HIV, insomnia, chronic illness) could contribute to the occurrence of SI (6–16). However, there is a significant gender difference in suicide deaths between men and women; the suicide death rate of men is higher than that of women (17).

After the coronavirus disease 2019 (COVID-19) pandemic was announced by WHO on March 11, 2020 (18), there were over 304 million confirmed cases and over 5.4 million deaths until January 9, 2022 (19). Each government has responded quickly and has adopted necessary positive measures after the pandemic, including advocating quarantine at home, wearing masks, social distancing, etc., (20, 21). However, subsequent repeated home isolation policy might have a profound impact on people's mental health. The prevalence of mental health problems has become much higher during the COVID-19 pandemic lockdown period, including suicide issues (22–26). However, the influence of COVID-19 on suicidal ideation and the gender differences in adolescents have not been well-studied.

We conducted a cross-sectional online survey to explore the prevalence of SI during the COVID-19 pandemic lockdown period in Chinese adolescents and to explore gender-specific correlated factors for SI.

## Methods

### Study Setting and Data Collection Procedure

This study was conducted in five cities/counties in Shandong Province, China, between June 9 and 29, 2020, when children and adolescents just went back to school after the COVID-19 pandemic lockdown (26). An online questionnaire survey (www.wjx.cn) was used to collect adolescents' general information, lifetime severe traumatic events, parenting style, and the specific status during the COVID-19 pandemic lockdown period. To control the research quality of the survey and to avoid repeated answers, the IP address of smartphones and computers were only accepted once. Two independent researchers checked the saved dataset separately.

The total numbers of the aimed primary and middle school students in target areas were 9,500 and 5,331 students (response rate = 56.12%), respectively, who completed the online survey. The inclusion criteria were as follows: (1) voluntary participation in the study and has signed the informed consent form (participants under the age of 18 need the consent of the guardian; (2) No major physical diseases or mental disorders; and (3) Completed the questionnaire completely without a large number of blank questions. Exclusion criteria were: (1) No informed consent has been signed; (2) Suffering from major physical diseases or mental disorders; (3) The questionnaire was not completed.

Among the 5,331 questionnaires, 5,175 were available (available rate = 97.07%). All surveys were conducted anonymously, and the privacy of the participants was respected. We obtained informed consent through the parents of the participants and the consent of the participants themselves.

The study protocol was approved by the Medical Ethics Committee of Jining Medical University (JNMC-2020-KY-004).

### Measurements

The Chinese version of the Egna Minnen av Barndoms Uppfostran (EMBU) was used to estimate the parenting style of the adolescents (27, 28). It has 66 questions including six types s of parenting styles by fathers (emotional warmth, punishment, favoring subject, over-interference, rejection, and overprotection) and five types of parenting styles by mothers (emotional warmth, punishment, favoring subject, rejection, over-interference, and overprotection). The EMBU demonstrated a high internal consistency in Chinese adolescents (28). In this study, if the score exceeded the mean score, we considered that the parenting to the participants presented the tendency in this dimension.

Severe lifetime traumatic events were measured by 13 specific questions. For example, “Have you ever experienced the deaths of your parents?”, “Have you ever experienced severe physical illness?”, “Have you ever been bullied at school?”, and “Have you ever had suicidal ideation or suicide attempt? etc.”? Participants needed to answer yes or no to each question.

Suicidal ideation was measured by asking “Have you ever had suicidal ideation during COVID-19 pandemic lockdown?” and the responses were categorized as “yes or no.”

Feeling depressed, feeling anxious, and having emotional emptiness were measured by the exact question as: “Have you ever had depressed mood during the COVID-19 pandemic lockdown period?”, “Have you ever had an anxious mood during the COVID-19 pandemic lockdown period?”, and “Do you often feel empty inside¿‘. In addition, we also investigated the participants' basic demographic characteristics, such as age, gender, grade, being an only-child, and the specific status during the COVID-19 pandemic.

### Statistical Analysis

The sample size needed in this study was calculated by the sample size calculation formula of epidemiological cross-sectional study. The formula was as follows: n$=\frac{Z_{1-\frac{\alpha }{2}}^{2}\pi \left(1-\pi \right)}{\delta^{2}}$ (where π was the population rate and δ was the allowable error). Based on Nock's findings, the prevalence rate (12.1%), α = 0.05, and δ = 0.02 (29). According to the principle of doubling the sample size of the cluster sampling, the number of the aimed minimum sample size was 2,025.

All analyses were performed with STATA 15.0 version (30). The statistical significance was set at the level of α = 0.05, and *P* < 0.05 was considered the statistical significance. The *t*-test or χ^2^ test is used to analyze differences in continuous variables or categorical variables to explore gender, urban and rural differences in demographic characteristics, or other variables. The prevalence of suicidal ideation during COVID-19 and associated 95% CI were estimated. Logistic regression was carried out to examine the relationships between the potentially related factors and the suicidal ideation. A gender-specific stepwise logistic regression was used to determine the final models. The odds ratio (OR) and their 95% CI were reported.

## Results

The sample population of our survey was randomly distributed in five counties in two cities, including the 8th grades. Finally, there were 5,175 available questionnaires. The characteristics of participants are displayed in Table 1. There were 2,673 (51.7%) males and 2,502 (48.3%) females. Among these, 3,813 (73.7%) were from rural and 1,362 (26.3%) were from urban. The age range was 9–19 years old and the mean (SD) of participants' age was 13.38 (1.56%). Most of the participants were junior high school students (60.2%), others were in primary school (25.9%), and senior high school (13.9%). There were 3,842 (74.2%) participants who lived with parents, 197 (3.8%) lived only with their father, and 816 (15.8%) lived only with their mother. Most of the participants have at least one sibling (78.9%). Besides, 1,346 (26%) felt depressed and 1,224 (23.7%) felt anxious. Among 5,175 students, 155 (3%) had suicidal ideation during the COVID-19 pandemic lockdown. Among 2,673 male and 2,502 female participants, there are 64 (2.39%) males with suicidal ideation, 2,609 (97.61%) males without suicidal ideation, 91 (3.64%) females with suicidal ideation, and 2,411 (96.36%) females without suicidal ideation. The characteristics of 5,175 participants by sex and suicidal ideation were displayed in Table 2.

**Table 1:** Characteristics of 5,175 participants and frequencies (%).

**Items**	**Variables**	**Categories**	***n***	**Frequency** **(%)**
Demographic characteristics				
	Age (years)	Mean ± SD	13.37 ± 0.02	
	Sex	Male	2,673	51.65
		Female	2,502	48.35
	One child	Yes	1,093	21.12
		No	4,082	78.88
	Residence	Urban	1,362	26.32
		Rural	3,813	73.68
	School level	Primary school	1,339	25.87
		Middle school	3,117	60.23
		High school	719	13.89
Specific status during COVID-19 pandemic lockdown				
	Feeling depressed	Yes	1,346	26.01
		No	3,829	73.99
	Feeling anxious	Yes	1,224	23.65
		No	3,951	76.35
	Insomnia	Yes	323	6.24
		No	4,852	93.76
	Quarreling with parents	Yes	343	6.63
		No	4,832	93.37
Parenting styles				
	Father emotional warmth	Yes	2,979	57.57
		No	2,196	42.43
	Father punishment	Yes	2,257	43.61
		No	2,918	56.39
	Father over-interference	Yes	2,767	53.47
		No	2,408	46.53
	Father favoring subject	Yes	2,794	53.99
		No	2,381	46.01
	Father rejection	Yes	2,156	41.66
		No	3,019	58.34
	Father overprotection	Yes	2,554	49.35
		No	2,621	50.65
	Mother	Yes	2,950	57.00
	emotional warmth	No	2,225	43.00
	Moher punishment	Yes	2,131	41.18
		No	3,044	58.82
	Mother	Yes	2,876	55.57
	over-interference and overprotection	No	2,299	44.43
	Mother favoring subject	Yes	2,852	55.11
		No	2,323	44.89
	Mother rejection	Yes	2,387	46.13
		No	2,788	53.87
Lifetime severe traumatic events				
	Being bullied at school	Yes	647	12.50
		No	4,528	87.50
	Being attacked	Yes	474	9.16
	by family/ teachers/strangers	No	4,701	90.84
	Previous suicidal ideation history	Yes	1,078	20.83
		No	4,097	79.17
	Previous suicide attempt history	Yes	610	11.79
		No	4,565	88.21
Personality trait				
	Emptiness inside	Yes	1,100	21.26
		No	4,075	78.74

**Table 2:** Characteristics of 5,175 participants by sex and suicidal ideation.

**Variables**	**Categories**	**Male**				**Female**			
		**With SI (%)**	**Without SI (%)**	**χ^2^/t**	***P***	**With SI (%)**	**Without SI (%)**	**χ^2^/t**	***P***
Demographic characteristics									
Age (years)	Mean ± SD	13.80 ± 1.72	13.37 ± 1.56	−2.18	0.030	13.25 ± 1.60	13.38 ± 1.54	0.78	0.437
One child	Yes	18 (28.13)	764 (29.08)			16 (17.58)	313 (12.98)		
	No	46 (71.87)	1,863 (70.92)	0.01	1.000	75 (82.42)	2,098 (87.02)	1.63	0.206
Residence	Urban	13 (20.31)	684 (26.22)			32 (35.16)	633 (26.25)		
	Rural	51 (79.69)	1,925 (73.78)	1.13	0.317	59 (64.84)	1,778 (73.75)	3.57	0.069
School level	Primary school	12 (18.75)	712 (27.29)			21 (23.08)	594 (24.64)		
	Middle school	39 (60.94)	1,543 (59.14)			57 (62.64)	1,478 (61.30)		
	High school	13 (20.31)	354 (13.57)	3.79	0.150	13 (14.28)	339 (14.06)	0.12	0.952
Specific status during COVID-19 pandemic lockdown									
Feeling depressed	Yes	42 (65.63)	679 (26.02)			65 (71.43)	560 (23.23)		
	No	22 (34.37)	1,930 (73.98)	49.73	<0.001	26 (28.57)	1,851 (76.77)	108.72	<0.001
Feeling anxious	Yes	39 (60.94)	590 (22.61)			67 (73.63)	528 (21.90)		
	No	25 (39.06)	2,019 (77.39)	50.99	<0.001	24 (26.37)	1,883 (78.10)	129.45	<0.001
Insomnia	Yes	29 (45.31)	130 (4.98)			44 (48.35)	120 (4.98)		
	No	35 (54.69)	2,479 (95.02)	181.61	<0.001	47 (51.65)	2,291 (95.02)	269.34	<0.001
Quarreling with parents	Yes	31 (48.44)	106 (4.06)			51 (56.04)	155 (6.43)		
	No	33 (51.56)	2,503 (95.94)	252.96	<0.001	40 (43.96)	2,256 (93.57)	285.70	<0.001
Parenting styles									
Father emotional warmth	Yes	20 (31.25)	1,576 (60.41)			14 (15.38)	1,369 (56.78)		
	No	44 (68.75)	1,033 (39.59)	22.07	<0.001	77 (84.62)	1,042 (43.22)	60.79	<0.001
Father punishment	Yes	48 (75.00)	1,284 (49.21)			65 (71.43)	860 (35.67)		
	No	16 (25.00)	1,325 (50.79)	16.61	<0.001	26 (28.57)	1,551 (64.33)	48.20	<0.001
Father over-interference	Yes	48 (75.00)	1,504 (57.65)			60 (65.93)	1,155 (47.91)		
	No	16 (25.00)	1,105 (42.35)	7.73	0.007	31 (34.07)	1,256 (52.09)	11.41	0.001
Father favoring subject	Yes	37 (57.81)	1,402 (53.74)			42 (46.15)	1,313 (54.46)		
	No	27 (42.19)	1,207 (46.26)	0.42	0.529	49 (53.85)	1,098 (45.54)	2.44	0.134
Father rejection	Yes	45 (70.31)	1,191 (45.65)			62 (68.13)	858 (35.59)		
	No	19 (29.69)	1,418 (54.35)	15.29	<0.001	29 (31.87)	1,553 (64.41)	39.95	<0.001
Father overprotection	Yes	45 (70.31)	1,410 (54.04)			57 (62.64)	1,042 (43.22)		
	No	19 (29.69)	1,199 (45.96)	6.67	0.011	34 (37.36)	1,369 (56.78)	13.43	<0.001
Mother emotional warmth	Yes	23 (35.94)	1,540 (59.03)			20 (21.98)	1,367 (56.70)		
	No	41 (64.06)	1,069 (40.97)	13.71	<0.001	71 (78.02)	1,044 (43.30)	42.79	<0.001
Mother	Yes	48 (75.00)	1,547 (59.29)			71 (78.02)	1,210 (50.19)		
over-interference and overprotection	No	16 (25.00)	1,062 (40.71)	6.40	0.014	20 (21.98)	1,201 (49.81)	27.19	<0.001
Mother rejection	Yes	46 (71.88)	1,225 (46.95)			75 (82.42)	1,041 (43.18)		
	No	18 (28.12)	1,384 (53.05)	15.56	<0.001	16 (17.58)	1,370 (56.82)	54.65	<0.001
Moher punishment	Yes	49 (76.56)	1,116 (42.78)			71 (78.02)	895 (37.12)		
	No	15 (23.44)	1,493 (57.22)	29.00	<0.001	20 (21.98)	1,516 (62.88)	61.89	<0.001
Mother favoring subject	Yes	38 (59.38)	1,452 (55.65)			46 (50.55)	1,316 (54.58)		
	No	26 (40.62)	1,157 (44.35)	0.35	0.611	45 (49.45)	1,095 (45.42)	0.58	0.455
Lifetime severe traumatic events									
Being bullied at school	Yes	25 (39.06)	326 (12.50)			38 (41.76)	258 (10.70)		
	No	39 (60.94)	2,283 (87.50)	38.65	<0.001	53 (58.24)	2,153 (89.30)	81.09	<0.001
Being attacked	Yes	24 (37.50)	243 (9.31)			35 (38.46)	172 (7.13)		
by family/ teachers/strangers	No	40 (62.50)	2,366 (90.69)	55.20	<0.001	56 (61.54)	2,239 (92.87)	113.40	<0.001
Previous suicidal ideation history	Yes	39 (60.94)	373 (14.30)			75 (82.42)	591 (24.51)		
	No	25 (39.06)	2,236 (85.70)	104.23	<0.001	16 (17.58)	1,820 (75.49)	150.53	<0.001
Previous suicide attempt history	Yes	31 (48.44)	199 (7.63)			65 (71.43)	315 (13.07)		
	No	33 (51.56)	2,410 (92.37)	132.29	<0.001	26 (28.57)	2,096 (86.93)	231.89	<0.001
Personality trait
Emptiness inside	Yes	33 (51.56)	439 (16.83)			78 (85.71)	550 (22.81)		
	No	31 (48.44)	2,170 (83.17)	51.84	<0.001	13 (14.29)	1,861 (77.19)	184.56	<0.001

The prevalence of suicidal ideation during the COVID-19 pandemic lockdown of all the participants was 3%. The prevalence of suicidal ideation in female participants (3.64 %) was higher than that in males (2.39%) (χ^2^ = 6.87, *P* = 0.009). There was no significant difference in the prevalence of suicidal ideation between adolescents in different grades (χ^2^ = 2.33, *P* = 0.312), one-child family structure (χ^2^ = 0.06, *P* = 0.801), and residence areas (χ^2^ = 0.61, *P* = 0.436).

The correlations between the related factors and the suicidal ideation during the COVID-19 pandemic lockdown were analyzed by univariate logistic regression (Table 3). Demographic characteristics (age), specific status during COVID-19 pandemic lockdown (feeling depressed, feeling anxious, insomnia and quarreling with parents), parenting styles (father's emotional warmth, father's punishment, father's over-interference, father's rejection, father's overprotection, mother's emotional warmth, mother's punishment, mother's over-interference and overprotection, and mother's rejection), and a lifetime of severe traumatic events (being bullied at school, being attacked by family/teachers/strangers, previous suicidal attempt history, previous suicide attempt history, and having emptiness inside) were associated with suicidal ideation in the participants during COVID-19 pandemic lockdown. Sex-specific results are in Table 3.

**Table 3:** The univariate logistic regression analysis results for suicidal ideation by gender during COVID-19 lockdown in 5,175 Chinese adolescents.

**Items**	**Factors**	**References**	**Male**	**Female**
			**OR (95% CI)**	**OR (95% CI)**
Demographic characteristics				
	Age	Continuous variable	1.19 (1.02–1.39)	0.95 (0.83–1.08)
	One child	Yes/No	1.02 (0.59–1.78)	0.70 (0.40–1.22)
	Residence	Rural/Urban	1.39 (0.75–2.58)	0.66 (0.42–1.02)
	Living arrangement	Single parent/With parents	0.88 (0.46–1.66)	1.48 (0.90–2.43)
		Without parents/ With parents	0.70 (0.22–2.30)	1.66 (0.78–3.54)
	School level	Middle school/ Primary school	1.50 (0.78–2.88)	1.09 (0.66–1.82)
		High school/ Primary school	2.17 (0.98–4.82)	1.08 (0.54–2.19)
Specific status during COVID-19 pandemic lockdown				
	Feeling depressed	Yes/No	7.81 (4.46–13.70)	40.53 (14.82–110.80)
	Feeling anxious	Yes/No	12.31 (6.85–22.13)	21.85 (11.26–42.41)
	Insomnia	Yes/No	15.80 (9.37–26.65)	17.87 (11.39–28.04)
	Quarreling with parents	Yes/No	22.18 (13.09–37.59)	18.56 (11.89–28.95)
Parenting styles				
	Father emotional warmth	Yes/No	0.30 (0.17–0.51)	0.13 (0.08–0.25)
	Father punishment	Yes/No	3.10 (1.75–5.48)	4.51 (2.84–7.16)
	Father over-interference	Yes/No	2.20 (1.25–3.90)	2.10 (1.35–3.27)
	Father favoring	Yes/No	1.18 (0.71–1.95)	0.72 (0.47–1.09)
	Father rejection	Yes/No	2.82 (1.64–4.85)	3.86 (2.47–6.06)
	Father overprotection	Yes/No	2.01 (1.17–3.46)	2.20 (1.42–3.39)
	Mother emotional warmth	Yes/No	0.39 (0.23–0.65)	0.22 (0.13–0.36)
	Moher punishment	Yes/No	4.37 (2.44–7.83)	6.01 (3.64–9.94)
	Mother over-interference and overprotection	Yes/No	2.06 (1.16–3.65)	3.52 (2.13–5.82)
	Mother favoring	Yes/No	1.16 (0.70–1.93)	0.85 (0.56–1.29)
	Mother rejection	Yes/No	2.89 (1.67–5.01)	6.17 (3.57–10.65)
Lifetime severe traumatic events				
	Being bullied at school	Yes/No	4.49 (2.68–7.52)	5.98 (3.87–9.25)
	Being attacked by family/ teachers/strangers	Yes/No	5.84 (3.46–9.86)	8.14 (5.19–12.76)
	Previous suicidal ideation history	Yes/No	9.35 (5.59–15.64)	14.44 (8.35–24.96)
	Previous suicide attempt history	Yes/No	11.37 (6.82–18.97)	16.63 (10.40–26.61)
Personality trait				
	Emptiness inside	Yes/No	5.26 (3.19–8.68)	20.30 (11.20–36.80)

The stepwise logistic regression results for suicidal ideation were shown in Table 4. Quarreling with parents (OR = 9.73, 95% CI: 5.38–17.59), insomnia (OR = 5.28, 95% CI: 2.81–9.93), previous suicide attempt history (OR = 3.68, 95% CI: 1.69–8.03), previous suicidal ideation history (OR = 2.81, 95% CI: 1.30–6.06), and feeling depressed (OR = 2.26, 95% CI: 1.22–4.18) were associated with the SI during COVID-19 lockdown in male participants. In female participants, having emptiness inside (OR = 4.39, 95% CI: 2.19–8.79), quarreling with parents (OR = 3.72, 95% CI: 2.16–6.41), insomnia (OR = 3.28, 95% CI: 1.85–5.80), feeling anxious (OR = 2.62, 95% CI: 1.46–4.70), previous suicide attempt history (OR = 2.53, 95% CI: 1.41–4.54), being bullied at school (OR = 2.03, 95% CI: 1.15–3.06), being attacked by family/teachers/strangers (OR = 1.84, 95% CI: 1.01–3.33), being the only child (OR = 0.48, 95% CI: 0.24–0.98), and father's emotional warmth (OR = 0.38, 0.20–0.72) were associated with SI during COVID-19 lockdown.

**Table 4:** The gender-specific stepwise logistic regression results for suicidal ideation during COVID-19 lockdown in 5,175 Chinese adolescents.

**Items**	**Factors**	**Male**	**Female**
		**OR (95% CI)**	**OR (95% CI)**
Demographic characteristics			
	One child	–	0.48 (0.24–0.98)
Specific status during COVID-19 pandemic lockdown			
	Feeling depressed	2.26 (1.22–4.18)	–
	Feeling anxious	–	2.62 (1.46–4.70)
	Insomnia	5.28 (2.81–9.93)	3.27 (1.85–5.80)
	Quarreling with parents	9.73 (5.38–17.58)	3.72 (2.16–6.41)
Parenting style			
	Father emotional warmth	–	0.38 (0.20–0.72)
Lifetime severe traumatic events			
	Being bullied at school	–	2.03 (1.15–3.60)
	Being attacked by family/ teachers/strangers	–	1.84 (1.01–3.33)
	Previous suicidal ideation history	2.81 (1.30–6.06)	
	Previous suicide attempt history	3.68 (1.69–8.03)	2.53 (1.41–4.54)
Personality trait			
	Emptiness inside	–	4.39 (2.20–8.79)

## Discussion

The prevalence of SI during the COVID-19 pandemic lockdown was 3% in this sample of Chinese children and adolescents. Females were more likely to have reported SI than males. Female adolescents, with emptiness from father's emotional warmth to the family, were at much higher risk of having SI during COVID-19 lockdown, while the male adolescents with feelings of depression, insomnia, quarreling with parents during COVID-19, suicidal ideation, or attempt history might associate these with suicidal ideation during COVID-19.

We found that there was an obvious sex difference in suicidal ideation in adolescents, which is consistent with other studies (31). Males and females had common factors for SI. However, there were sex-specific factors associated with SI. Feeling depressed was associated with SI in males rather than in females. Feeling anxious was only associated with SI in females rather than in males. Therefore, given the sex difference, we should pay more attention to different symptomatic manifestations in boys and girls to prevent the occurrence of suicidal behaviors during natural disasters like the COVID-19 pandemic. However, only a few studies are exploring the relationship between mood disorders and suicidal ideation by sexes, and more future studies are needed.

The prevalence of suicidal ideation (3%) in adolescents was relatively low compared with that in other studies (12.7%) (10). This may be similar to the result of other studies indicating that the suicide rate in adolescents during the COVID-19 pandemic was not raised, and Tanaka's study showed that the suicide rate declined by 14% in the first 5 months in Japan (22, 32). There are several possible reasons for that. First, home isolation may reduce the chance of negative interpersonal communication with their peers, which may improve the mental health of adolescents (33). Second, according to the results of this study, some adolescents had poor relationships with parents (such as quarreling with parents). This study was conducted when the participants just returned to school and spent more time at school, which might have reduced the conflicts with parents. Third, it is possible that the COVID-19 pandemic lockdown did not have a dramatic impact on suicidal ideation (23–26). Other studies also showed that there was no obvious change of suicidal ideation in adolescents during the COVID-19 pandemic lockdown (32).

Some specific behaviors (quarreling with parents, insomnia, etc.) during COVID-19 may be associated with the onset of SI in adolescents during COVID-19 pandemic lockdown and had negative impacts on adolescents' mental health. Because of the COVID-19 pandemic, it was difficult to seek professional help. In this context, telepsychiatry is a promising way of mental health service delivery to address the issues to enhance the children and youth's ability to cope with stress, alleviate depressed mood, and reduce their risk of suicide (34, 35). Given the huge gender difference for suicidal ideation in adolescents, independent predictive models based on gender may be necessary to identify the high-risk individuals for suicidal ideation in adolescents. A finding from our result is that a good fatherly emotional warmth may be an important factor to prevent the occurrence of suicidal ideation in female adolescents.

A suicide prevention system, based on the school-family-community joint mechanism for children and adolescents, should be built (36). For the entire society, the suicide prediction and intervention systems need to be established to identify the high-risk individuals through the system and intervene promptly. In addition, a developmentally-sequenced upstream suicide prevention approach is extremely important (36). Firstly, the parents and the schools should try to eliminate risk factors of suicide such as school bullying and parental abuse. Secondly, we should also pay more attention to strengthening the psychological and behavioral education in children and adolescents to improve mental health, and in minimizing the impact of possible negative events.

Globally, we are still suffering from the COVID-19 pandemic (19) and Murray said that by March 2022, more than 50% of people in the world will be infected with Omicron, and 80–90% of them will be asymptomatic (37). At that time, the global immunization level will be at the highest level in history due to continuous vaccination and because of the immunity caused by virus infection. In a few weeks or months, the level of COVID-19 spread will decrease (37). Each government should publicize epidemic-related policies and promote scientific knowledge on COVID-19 to reduce public panic, especially for adolescents, and reduce the negative impact of rumors (which may increase the risk of suicide), scientifically and correctly arrange online classes, and control the influential impact of home isolation on adolescents (38, 39). In the post-pandemic era, the beginning of normal life is also an important period to deal with its sequelae and prevent the potential increase in the suicide rate (23). Changes in the financial conditions of the family (e.g., being laid off), changes in adolescents' social relationships due to home isolation, and discomfort after returning to school may also worsen the mental health of adolescents (40–42). During this period, the school can play an important role in maintaining and improving mental health and preventing suicide in students (43). Adolescents spend more time at school than with their families. Therefore, the school-related department needs to adjust the adolescents' mindset and learning habits to mitigate the adverse impact of the COVID-19 pandemic.

## Limitations

There were several limitations in this study. First, suicidal ideation and exposures were collected by self-report in this cross-sectional study. Therefore, recall and reporting biases are possible. Second, there were limited correlated factors collected in this study, and some other exposures, such as the history of mental disorders and biological factors, were not collected (44). Third, the participants in this study were only sampled from two cities in Shandong Province, China. Furthermore, because of the relatively low response rate (56%), this sample may not be representative of the Chinese adolescent population. Finally, in this cross-sectional study, the causal relationships between exposures and suicidal ideation could not be inferred. Cohort studies were needed.

## Implications For Future Research

In our research, a specific status during COVID-19 pandemic lockdown, lifetime severe traumatic events, and parental rearing styles could influence the prevalence of suicidal ideation among adolescents, and there was also a considerable gender difference. At present, there are few studies on the suicide behavior of adolescents during and after the COVID-19 pandemic. Although most of the regions in China have lifted the lockdown measures and returned to normal life, some cities may have to re-implement restrictive measures because of the recurrence of COVID-19. Many countries are still suffering from COVID-19. Therefore, our study generated new knowledge for informing the public health policies and raising awareness about adolescent mental health.

## Conclusions

Female adolescents, with a feeling of emptiness from their father's emotional warmth to the family, were at much higher risk of having SI during COVID-19 lockdown. Identifying the predictors of suicide behaviors of different genders is conducive to preventing suicide and is a more effective identification of suicide high-risk groups. Therefore, we have to specify a suicide prevention policy and interventions for adolescents in the pandemic crisis based on gender gaps.

## Data Availability Statement

The raw data supporting the conclusions of this article will be made available by the authors, without undue reservation.

## Ethics Statement

The studies involving human participants were reviewed and approved by Research Ethics Committee in Jining Medical University. Written informed consent to participate in this study was provided by the participants' legal guardian/next of kin.

## Author Contributions

DL, JW, and YL contributed to the study design. LL, DL, SY, and JZhu did the online survey, data collection, and logical check. MC, JZha„ YL, and DL analyzed the data. JZhu, BL, FH, and LL wrote this manuscript. DL, YL, and JW revised the manuscript and did the English revision. All authors reviewed and approved the manuscript.

## Funding

This study was funded by the Research Fund for Lin He's Academician Workstation of New Medicine and Clinical Translation in Jining Medical University to JZhu (Grant Number: JYHL2019MS05, JYHL2021MS14), Taishan Scholars Project of Shandong Province (tsqn201909145), and Key Research and Development Plan of Jining (Grant Number: 2019SMNS033) to YL, and Innovative Training Program for College Students in Jining Medical University to DL (Grant Number: cx2020009).

## Conflict of Interest

The authors declare that the research was conducted in the absence of any commercial or financial relationships that could be construed as a potential conflict ofinterest.

## Publisher's Note

All claims expressed in this article are solely those of the authors and do not necessarily represent those of their affiliated organizations, or those of the publisher, the editors and the reviewers. Any product that may be evaluated in this article, or claim that may be made by its manufacturer, is not guaranteed or endorsed by the publisher.
